# The Influence of Tactile Cognitive Maps on Auditory Space Perception in Sighted Persons

**DOI:** 10.3389/fpsyg.2016.01683

**Published:** 2016-11-01

**Authors:** Alessia Tonelli, Monica Gori, Luca Brayda

**Affiliations:** ^1^Unit for Visually Impaired People, Science and Technology for Children and Adults, Istituto Italiano di TecnologiaGenova, Italy; ^2^Robotics, Brain and Cognitive Sciences Department, Istituto Italiano di TecnologiaGenova, Italy

**Keywords:** cognitive maps, space perception, bisection, calibration, auditory perception, non-informative touch, multisensory

## Abstract

We have recently shown that vision is important to improve spatial auditory cognition. In this study, we investigate whether touch is as effective as vision to create a cognitive map of a soundscape. In particular, we tested whether the creation of a mental representation of a room, obtained through tactile exploration of a 3D model, can influence the perception of a complex auditory task in sighted people. We tested two groups of blindfolded sighted people – one experimental and one control group – in an auditory space bisection task. In the first group, the bisection task was performed three times: specifically, the participants explored with their hands the 3D tactile model of the room and were led along the perimeter of the room between the first and the second execution of the space bisection. Then, they were allowed to remove the blindfold for a few minutes and look at the room between the second and third execution of the space bisection. Instead, the control group repeated for two consecutive times the space bisection task without performing any environmental exploration in between. Considering the first execution as a baseline, we found an improvement in the precision after the tactile exploration of the 3D model. Interestingly, no additional gain was obtained when room observation followed the tactile exploration, suggesting that no additional gain was obtained by vision cues after spatial tactile cues were internalized. No improvement was found between the first and the second execution of the space bisection without environmental exploration in the control group, suggesting that the improvement was not due to task learning. Our results show that tactile information modulates the precision of an ongoing space auditory task as well as visual information. This suggests that cognitive maps elicited by touch may participate in cross-modal calibration and supra-modal representations of space that increase implicit knowledge about sound propagation.

## Introduction

Several studies show that vision is essential in the domain of space perception influencing also other sensory modalities. It is well known that auditory space perception is modulated by visual inputs. When an auditory and visual stimuli are simultaneously presented although in two different space locations, the auditory stimulus is localized toward the location of the visual stimulus. This phenomenon is known as Ventriloquist effect (Bertelson and Radeau, 1981; Warren et al., 1981). Unlike the visual system, the auditory system cannot rely on a retinotopic organization of space in the inner ear. Specifically, the brain has to infer the direction of sound sources by taking into account the relative intensity of sound received at each ear as well as the time delay between arrival at the two ears in the superior olivary complex (Middlebrooks and Green, 1991). For this reason the auditory system is normally less accurate and reliable in spatial representation, compared with the visual system. Interestingly, vision can interact with audition even when a visual stimulus is not provided during an auditory task (Jackson, 1953; Shelton and Searle, 1980; Tabry et al., 2013). We recently demonstrated in sighted people that performance in auditory space bisection tasks is calibrated by short-term environmental observation only in a reverberant room, meaning that vision helps to construct complex auditory cognitive maps (Tonelli et al., 2015) thanks to a mental representation of the environment and not by direct visual information. Along with that, several studies have demonstrated, at a perceptual level, that auditory space perception can also be biased by tactile stimuli. Similarly to the audio-visual Ventriloquist effect, auditory localization seems biased toward the side of the concurrent tactile stimulus in bimodal tasks (Caclin et al., 2002; Bruns and Röder, 2010a,b; Bruns et al., 2011). Specifically, tactile stimulation influences the auditory cortical activity through higher areas assigned to multimodal association (Bruns and Röder, 2010a).

In the present study, we investigate whether it is possible to use touch as substitute of vision to modify, and possibly to improve, auditory spatial representations through the creation of a mental representation of the environment. We tested whether the construction of a cognitive map of a room through touch, can *indirectly* influence the perception of a complex auditory task (i.e., auditory spatial bisection task) in sighted people.

The hypothesis of this study is that haptic three-dimensional knowledge of an environment helps to build more precise auditory cognitive maps. This would match our previous results where vision calibrates the auditory modality (Tonelli et al., 2015). We supposed that spatial information obtained by exploring a 3D map would be poorer than that gained by visual observation. However, we wondered if, still, tactile information would be ‘enough for space’, meaning that essential information about the perimeter of the room, the kind of objects and their spatial relation would constitute sufficient knowledge to emulate the contribution of vision in auditory space perception (Pasqualotto et al., 2013).

To test this hypothesis, we tested a group of blindfolded sighted people in an auditory space bisection task and allowed them to explore with the hands a 3D tactile model of the room between the first and the second execution of the auditory task.

We recall that mental representation is an internal cognitive idea that represents external reality or else a mental process that makes use of such idea: “*a formal system for making explicit certain entities or types of information, together with a specification of how the system does this*” (Marr, 1985).

In our case we wanted to evaluate the mental spatial ability of the participants, through mental manipulation of objects in space. In addition, studies have demonstrated that the inter-personal variability in performing mental manipulations (Parsons et al., 2004; Guillot et al., 2007) is quite high. We therefore hypothesized that the ability in representing or manipulating an object could possibly predict auditory space bisection performance, when supported by additional haptic or visual knowledge of the room.

We administered to each participants two mental rotation questionnaire: the paper folding test (PFT) and the mental rotation test (MRT). The PFT requires participants to mentally perform complex spatial manipulations (Ekstrom et al., 1976) of a 2D item. Instead, the MRT evaluates the ability of mentally rotating a 3D object (Shepard and Metzler, 1971). The hypothesis was that PFT may predict an improvement obtained after the exploration of the tactile map – more similar to elicit mainly bi-dimensional representation, while the MRT would predict an improvement obtained after visual observation, which is more likely to elicit three-dimensional representations.

## Materials and Methods

### Participants

Twenty sighted participants (13 females and 7 males, with an average age of 28.5, *SD* = 7) were recruited to participate in the experiment. All participants gave written informed consent before starting the test. The study was approved by the ethics committee of the local health service (Comitato Etico, ASL 3, Genova).

### Apparatus and Stimuli

**FIGURE 1:**
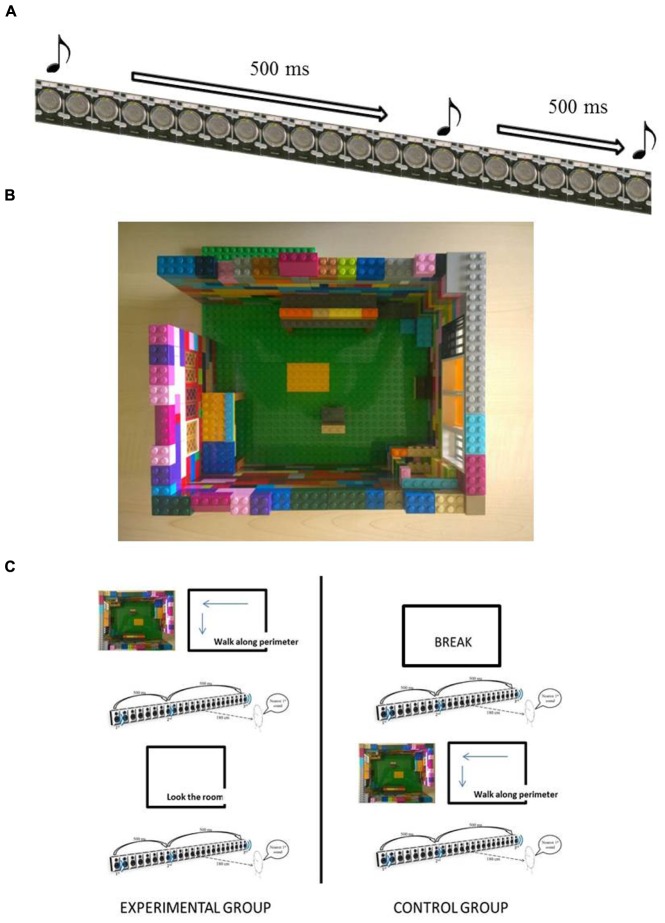
**Set-up and procedure. (A)** Space bisection task. **(B)** 3D model of the room seen from the above. **(C)** Left part, shows the procedure for the experimental ground and the right part shows the procedure for the control group.

Stimuli were delivered with scripts exploiting the Psychophysics 3.08 (Brainard, 1997) tool and Matlab (R2009a, The Mathworks, Natick, MA, USA). The acoustic stimuli for the auditory space bisection task were pink noise bursts, lasting 75 ms each. The sounds were produced by a linear array of 23 loudspeakers, 161 cm long and spanning ±25° of visual angle (see **Figure 1A**). The participants sat 180 cm from the center of the array. The auditory space bisection task consists in playing three consecutive sounds (duration of 75 ms) with an interval between each sounds of 500 ms. The first stimulus came always from the loudspeaker to the left (-25°) and the third stimulus from the loudspeaker to the right (+25°). The second stimulus came from an intermediate position, which was determined by QUEST (Watson and Pelli, 1983), an adaptive algorithm which, based on the current estimation of the participant, estimates the best stimulus value to be presented in the subsequent trial. The proportion of ‘rightward’ responses was calculated for each speaker distance. Gaussian functions by means of the Maximum Likelihood method were used to estimate both the accuracy, i.e., the bias in localize the center of the array, and the standard deviation. The standard deviation of the fit was taken as an estimate of the threshold, indicating the precision of the task, i.e. the reliability with which the task is performed.

The room size was 4.2 m × 3.0 m × 3.2 m (height) and the 3D reproduction of the room was made by bricks of Lego© on a scale 1:15 (see **Figure 1B**). Therefore, the space of the room was represented by a 30 × 22 Lego dots matrix (excluding the walls, which were two Lego dot thick), i.e., a tactile map of 27 cm × 20.7 cm. The walls of the map were 10 Lego bricks high.

The bricks represented the perimeter of the room, the relevant openings (door, window) and the main objects located in the room (two tables, the chair hosting the subject), including a tactile representation of the loudspeaker array. A small model of a man, representing the subject, gave hint about his/her correct position and orientation inside the room and with respect to the objects. We respected the approximate relative proportions of all objects in the room.

Each participant was given two questionnaires evaluating mental manipulation ability: a PFT and a MRT. The PFT required participants to mentally perform complex spatial manipulations (Ekstrom et al., 1976). For each item on the PFT, the drawings depicted two or three folds in a square sheet of paper. The last drawing of folded paper showed a hole punched in it. Participants selected one of five drawings showing how the punched paper would look like when fully reopened. It was composed by 20 questions with scores ranging from 0 to 20. The MRT, instead, is composed by figures provided by Shepard and Metzler (1971), modified by Peters et al. (1995). The participants had to rotate the figures both around the horizontal and vertical axis in order to obtain the correct solution. The score was calculated by giving one and only one point for each correctly solved problem. A correct solution consists in identifying those two stimuli from a group of four, which represent rotated versions of the target stimulus.

### Procedure

The sample of participants was randomly assigned in one of two groups (see **Figure 1C**): an experimental group and a control group.

Both groups performed an auditory space bisection task. The participants verbally reported whether the second sound was spatially closer to the first sound (produced by the first speaker on the left, number 1) than the last sound (produced by the last speaker on the right, number 23).

Both groups performed the task three times. All the participants were blindfolded before entering the room, so that during the first execution of the auditory task, they had neither knowledge of the room nor of the setup used to deliver the acoustic stimuli. The experimental group, blindfold on, explored with both hands the 3D tactile model of the room to understand the structure of the room, the disposition of the main objects inside the room, their own relative position with respect to the room and the objects when performing the auditory task. After that, each participant was led counterclockwise along the perimeter of the actual room. The participant had the chance to touch the walls and the acoustic stimulation setup. The participant of the experimental group then performed the auditory task a second time. Following that, the blindfold was removed for 1 min – allowing visual observation. Finally, the participant performed the auditory task a third time. Instead, the control group, after the first execution of the task, had a break of 5 min, keeping the blindfold on, then performed the auditory task a second time. As a last action, the control group followed the same procedure of the experimental group for the tactile exploration and navigation through the environment, then performed the task a third time. Each subject performed 80 trials of the auditory task per repetition, for a total of 240 trials.

At the end of the auditory space bisection task the participants of the experimental group were administered the PFT and the MRT, in random order. The PFT was administered in two parts of 10 questions each and they had 3 min to complete each part with a break of 1 minute between the first and the second parts. To complete the MRT the participants had 10 min.

## Results

We ran a Lilliefors (Kolmogorov-Smirnov) test to check the normality of the sample. Results showed that both the experimental and control groups were not normally distributed for the precision in the first execution of the task (experimental group, *D* = 0. 279, *p* < 0.03; control group, *D* = 0. 277, *p* < 0.03; for more information, see Supplementary Materials). We used non-parametric statistical analysis. The failure in respecting criteria for normality is due to the presence of two outliers performances: participant 3 in the experimental group and participant 6 in the control group.

**FIGURE 2:**
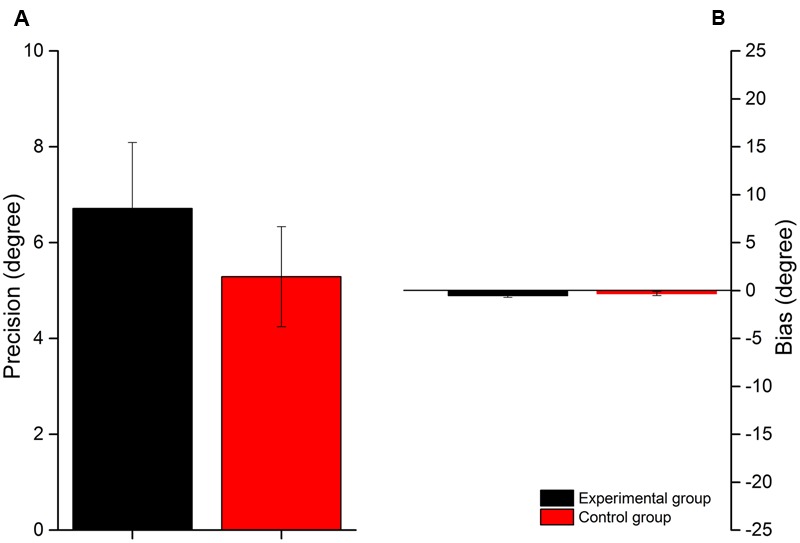
**First execution in control group and experimental group.** Comparison between first execution in experimental group (black bars) and control group (red bars) for the precision **(A)** and the bias respect to the center of the loudspeaker array **(B).**

To see if the two samples were comparable we performed a Wilcoxon-test analysis (two-paired sample) between the first execution of the two groups. The results (**Figure 2**) revealed no significant difference between the first execution of the experimental group (black bars) and the control (red bars) for both precision (*W* = 65.5, *p =* 0.26) and bias (W = 41.5, *p =* 0.54), suggesting that the two groups are comparable, even if the control group is slightly more precise as compared to the experimental group.

We decided to normalize the results of the post-touch and post-vision, in the experimental group, and, second execution and post-touch, in the control group, by the performance of each participant in the first execution to avoid biases. For both precision and accuracy (bias), we computed a relative improvement: we subtracted to each performance that obtained in the first execution, then we divided it again for the first execution.

**FIGURE 3:**
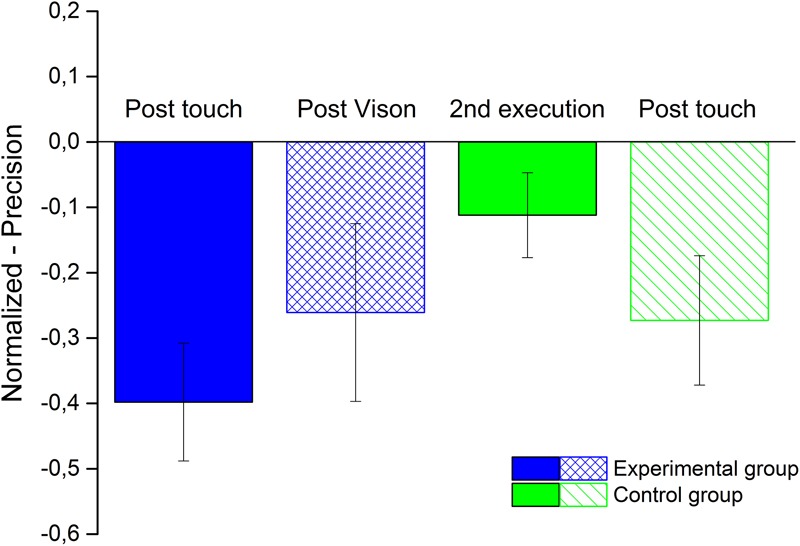
**Space Bisection precision.** The bar plot shows the average precision of both groups obtains normalizing the performance of each participants by their performance in the first execution. The blue bars represent the experimental group for the condition post-touch (filled bar) and post-vision (lined bar). The green bars represent the control group for the condition second execution (filled bar) and post-touch (lined bar).

After that, we analyzed the precision in both the experimental and control groups, performing a one-sample Wilcoxon test for each condition of the experimental group, post-touch and post-vision conditions, and control group post-touch and second execution. In the post-touch condition, we had nine participants, instead of 10. As showed in **Figure 3**, for the experimental group, we found a significant improvement in precision for the experimental group (blue bars) in post-touch condition (filled blue bar -*V* = 1, *p* < 0.01), but not in the post-vision (lined blue bar: *V* = 9, *p* = 0.06), even if there is a trend. For the control group (green bars), we found a significant improvement for the post-touch condition (lined green bar: *V* = 3, *p* < 0.02) and not for the second execution (filled green bar: *V* = 16.5, *p* = 0.28).

**FIGURE 4:**
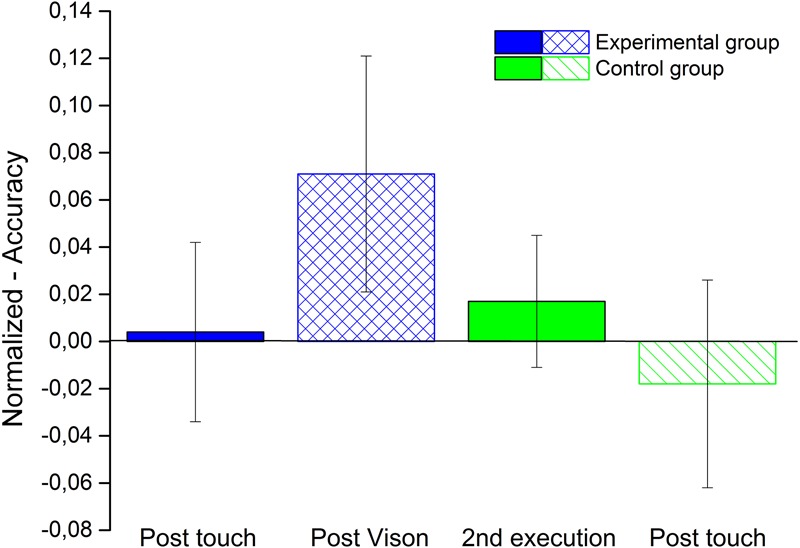
**Space Bisection accuracy.** The bar plot shows the average accuracy of both groups obtains normalizing the performance of each participants by their performance in the first execution. The blue bars represent the experimental group for the condition post-touch (filled bar) and post-vision (lined bar). The green bars represent the control group for the condition second execution (filled bar) and post-touch (lined bar).

On the contrary for the bias in performing the task, as showed in **Figure 4**, we did not found a significant improvement for accuracy in any condition for both control group (green bars - 2nd execution, *V* = 32.5, *p* = 0.65; post-touch *V* = 21, *p* = 0.91) and the experimental group (blue bars - post-touch, *V* = 27, *p* = 1; post-vision *V* = 39, *p* = 0.27).

Concerning the questionnaires, the average scores for the PFT was 62% of correct responses (*SD* = 16.5) and for the MRT was 51.7 % of correct responses (*SD* = 19). We computed a correlation between the percentage of correct responses in each questionnaire and the performance after tactile or visual information for both precision and accuracy. Thus, we computed a non-parametric Spearman correlation (RHO). After a Bonferroni correction for multiple comparisons, we found a negative and highly significant correlation only between the precision of post-touch condition (ρ_(20)_ = -0.83, *p* < 0.01) and PFT. For the other results, see **Table 1**.

**Table 1:** Results of the correlation between two questionnaires about spatial abilities and precision/accuracy of the auditory space bisection tasks in two conditions (post-touch and post-vision).

	Post-touch	Post-vision	Post-touch	Post-vision
	Accuracy	Accuracy	Precision	Precision
PFT	ρ = -0.18, *p* = 0.61	ρ = 0.15, *p* = 0.67	ρ= -0.83, *p* < 0.01	ρ = -0.73, *p* = 0.02

MRT	ρ = 0.07, *p* = 0.85	ρ = 0.14, *p* = 0.7	ρ = -0.33, *p* = 0.35	ρ = -0.55, *p* = 0.1

## Discussion

Although previous studies (Bruns and Röder, 2010b; Gori et al., 2014b) demonstrated how *direct* tactile stimuli can influence auditory perception, this is the first study showing that the sense of touch, through active exploration of a surrounding environment and of its 3D map, can *indirectly* influence complex audio-spatial tasks that are known to benefit from previous environmental knowledge. This work contributes to argue that spatial representations are unlinked to specific sensory modalities and that cross-modal calibration therefore contributes to build supra-modal mental representations.

Recent studies highlighted the importance of vision during development (Gori et al., 2012) showing that during childhood vision calibrates the other senses to process spatial information. When this calibration cannot take place, the non-visual modalities, especially audition, cannot properly encode some spatial information (Gori et al., 2014a; Finocchietti et al., 2015; Voss et al., 2015) that required a metric representation of space, while other auditory tasks are preserved (Lessard et al., 1998; Voss et al., 2004). In our previous study (Tonelli et al., 2015) we found that, in a reverberant room, the absence of knowledge of the environment leads to a decrease in precision of a complex auditory task, while no decrease occurs if the same task is performed in an anechoic chamber. This “*impairment*” is recovered after a brief observation of the room. The idea is that the person during the visual observation of the room has the chance to create a mental representation of the space. Thanks to this representation the auditory system becomes able to compensate the noise produced by the reverberation. However, spatial knowledge does not come from vision only. Similar findings in which cognitive maps are developed from other modalities can shed light on the underpinnings of auditory spatial processing. For example, we have shown that touch helps to develop cognitive maps of surroundings in absence of vision (Campus et al., 2012) by eliciting the known phenomenon of sensory substitution (Bach-y-Rita and Kercel, 2003). However, when comparing persons with different degrees of visual disabilities, vision modulates the extent to which tactile information builds up abstract mental models (Brayda et al., 2013, 2015). On the other hand we have also found that brain regions deputed to tacto-spatial processing are similar to those elicited by audio-spatial processing (Leclerc et al., 2005; Campus et al., 2012).

In the present study, we investigated two points: (i) whether cognitive maps created by touch could influence space auditory perception with the same efficiency of maps generated by visual information, and (ii) whether the ability to mentally manipulate an object could predict auditory space auditory perception, when supported by additional haptic or visual knowledge of the room.

Contrarily to the study mentioned above, in the present study we allowed participants to construct a cognitive map by exploring with their hands a 3D model of the room and by being led along the perimeter of the real room between two executions of the space bisection task.

We found that tactile exploration significantly increases precision in a space bisection task. One could argue that the improvement might be due to a learning process and not to the tactile exploration. We have shown that this is not the case, because a control group, who performed the task twice and without any feedback on the structure of the room, did not show significant improvement in precision after the second task execution, but exhibited a significant improvement in the third execution, after tactile exploration of the 3D model of the room and by being led along the perimeter of the real room. The smaller magnitude of the accuracy improvement of the control group after touch, as compared to the experimental group, may be partially due to a learning effect. In fact, the sum of *s* the improvement of the control group after the second execution (-0.08) and the post-touch (-0.27) equals that of the experimental group after the tactile exploration (experimental group = -0.39; sum of the control group = -0.35).

Therefore we maintain that, in agreement with our previous study (Tonelli et al., 2015), the information obtained by touch, combined with vestibular feedback during navigation, are sufficient cues to create a mental representation of the space that helps to improve the understanding of room acoustics. Since observing the room a does not further increase auditory precision compared to touch a 3D model of the room, we assert that touch gives sufficient cues to create a mental representation of space, even if vision is generally more suitable to address space perception and representation.

The results obtained in this study may appear not surprising, because previously studies demonstrated that passive tactile stimuli can directly influence auditory localization. For example, Gori et al. (2014b) demonstrated that a direct tactile feedback interacts with auditory spatial localization system improving the precision, if it is presented right after the auditory stimuli and in a congruent position. On the other hand, if it is present a spatial discrepancy between the tactile stimuli and the auditory stimuli, the auditory localization seems biased concurrent with the tactile stimuli in bimodal tasks (Caclin et al., 2002; Bruns and Röder, 2010a,b; Bruns et al., 2011). An explanation is that an incongruent condition may cause a cortical remapping of the auditory spatial representation, which tends to be more similar to the tactile spatial representation. What differs in this study is that the influence of tactile information on a complex auditory task is *indirect* and resides in the mental map create thanks to tactile information. This is visible in three main aspects. First, the tactile stimuli are not passively delivered on the human body, but are actively generated from spontaneous haptic exploration. The role of active exploration as compared passive stimulation is known to be important when building cognitive maps (Heller and Schiff, 1991). Second, comparing to previous studies, tactile feedback does not occur simultaneously with the auditory spatial task as in Gori et al. (2014b): here the spatio-tactile and audio-tactile information are not linked to the same stimulus, but are just a mean to create a mental representation of the environment. Third, in our experiment tactile and audio feedbacks do not necessarily share the same frame of reference, since haptic exploration involved navigation and consequent stimulation of the vestibular system, while acoustic stimulation was a task to be achieved while seating. In fact, tactile stimuli initially have egocentric reference frame and then are remapped into external coordinates influencing the auditory space perception (Bruns and Röder, 2010a). The cognitive map obtained through touch is the additional piece of information that improves auditory precision.

The choice of space bisection deserves further explanation. Performing space bisection requires establishing a specific ordering relation between the three sound sources and take a decision based on these relations. This operation may require Euclidian representation of space (Gori et al., 2014a) and involves more spatial processing, possibly related to cues linked to the room structure.

The mental representation of space allows to interact with objects, to move into the environment and is based onto two frame of reference: allocentric and egocentric (Klatzky, 1998). The first is based on external salient landmarks in the environment; the second refers to coordinates anchored to the body. Starting from these two spatial coding modes we are able to create cognitive maps of space based on two different perspectives: *survey* and *route*. The *’survey’* prospect provides a holistic view of the environment, preserving the information on the position of the objects and the Euclidean distances between them (Shelton and McNamara, 2004). One inevitable limitation of this study is that we could not counterbalanced across the participants of the experimental group the tactile and visual condition, because, otherwise, we would not have been able to assess the effect that the mental representation, built through tactile exploration, would have on the space bisection task.

Given the importance of the mental representation to perform the task and the nature of the space bisection, we decided to see whether there was a correlation between the results in the space bisection task and two mental rotation questionnaires: PFT and MRT. Mental rotation and mental folding have in common underlying cognitive process (Pellegrino et al., 1984; Wright et al., 2008). However, these two abilities differ, because mental folding is a non-rigid spatial transformation ability where the features of the manipulated object change. Instead, mental rotation involves a rigid manipulation, the object itself results unchanged, rather its spatial orientation differs. (Harris et al., 2013).

Our results show a significant negative correlation between the percentage of correct responses in the PFT and the precision of the space bisection task for post tactile exploration, meaning that the higher precision in the bisection task, the greater the ability to mentally manipulate a folded object. This correlation is much weaker with the MRT. One possible explanation could be that to solve a mental folding test, analytic strategy (Kyllonen et al., 1984) is needed, that helps to perform a non-rigid spatial transformation of the features of the manipulated object. This may in principle differ from MRT that requires, instead, a single rotation. The same strategy involved in PFT could be applied to perform the space bisection task. After having acquired spatial information through tactile or visual exploration, a common need to put in relationship the coordinates of the three sounds (or of the facets on the paper) may appear, which establishes a specific ordering relation between the map of sound sources or, alternatively, the map of the facets.

Our results show that a mental representation of the environment helps to perform complex spatial auditory tasks and that this representation can be create using both visual and tactile information. Moreover, we found that it is possible to correlate the precision in the space bisection task based on the results obtained in the PFT.

## Author Contributions

AT, MG, and LB have contributed in designing the work. Data acquisition and analysis was provided by AT. AT, MG, and LB have contributed in drafting the work and gave final approval to the manuscript.

## Conflict of Interest Statement

The authors declare that the research was conducted in the absence of any commercial or financial relationships that could be construed as a potential conflict of interest.
