# Supplementary figures and images for: The Influence of Tactile Cognitive Maps on Auditory Space Perception in Sighted Persons
Source: Front Psychol. 2016 Nov 1;7:1683. doi: 10.3389/fpsyg.2016.01683 (PMC5088781; doi:10.3389/fpsyg.2016.01683)

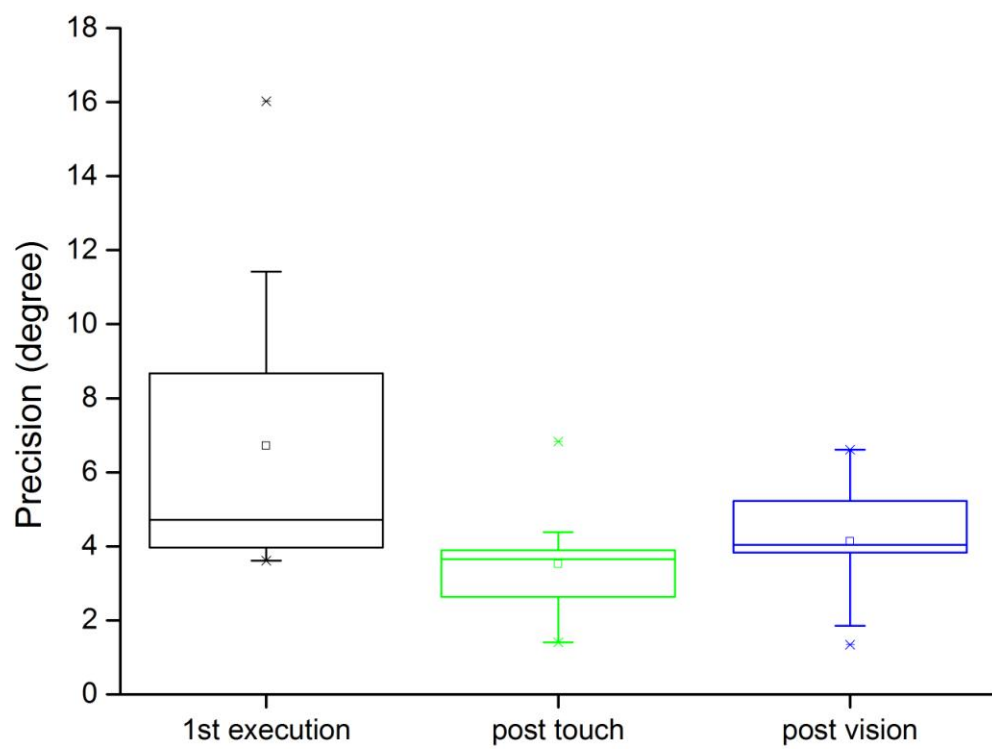

Image 1. Blox plot representing the data of the experimental group.

Supplement: Supplementary file 1 [file Image_1.PDF]

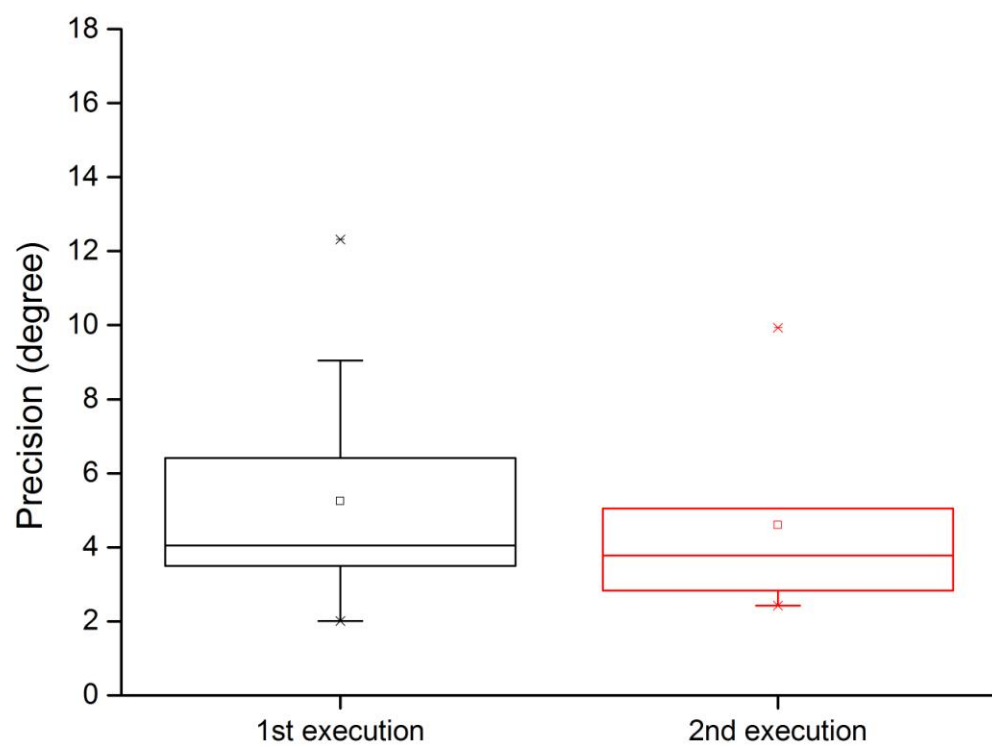

Image 2. Blox plot representing the data of the control group.

Supplement: Supplementary file 2 [file Image_2.PDF]
